# Optimal conditions of mycelia growth of *Laetiporus sulphureus* sensu lato

**DOI:** 10.1080/21501203.2014.957361

**Published:** 2014-09-16

**Authors:** Thatsanee Luangharn, Samantha C. Karunarathna, Kevin D. Hyde, Ekachai Chukeatirote

**Affiliations:** ^a^ School of Science, Mae Fah Luang University, Chiang Rai57100, Thailand; ^b^ Institute of Excellence in Fungal Research, Mae Fah Luang University, Chiang Rai57100, Thailand

**Keywords:** edible mushroom, *Laetiporus sulphureus*, optimal conditions

## Abstract

*Laetiporus sulphureus* is an edible wood-rotting basidiomycete, growing on decaying logs, stumps, and trunks of many deciduous and coniferous tree species. This fungus produces relatively large striking yellowish or orange-coloured bracket-like fruitbodies. *L. sulphureus* is widely consumed as a nutritional food because of its fragrance and texture. In this study, two *L. sulphureus* strains, MFLUCC 12-0546 and MFLUCC 12-0547, isolated from Chiang Rai, Thailand, were investigated for optimal conditions of mycelia growth. Potato dextrose agar and malt extract agar were observed as the favourable medium for mycelia growth. The optimum pH and temperature for the mushroom mycelia were 6–8 and 25–30°C, respectively.

## Introduction

*Laetiporus sulphureus* (Bull.: Fr.) Murrill. sensu lato belonging to *Basidiomycotina, Aphyllophorales, Polyporaceae* was first reported by Murrill in 1904 and typified by *Boletus sulphureus* Bull. (Ryvarden and Gilbertson 1993). This is recognized as a mushroom with soft consistency, distinctive striking citric-yellow to pale orange bodies, and grows as single or imbricate in clusters (Ryvarden and Johansen 1980; Wiater et al. 2013). This fungus is saprobic and parasitic; it often causes red-brown cubical heart rot with thin areas of white mycelium visible in the cracks of wood and occurring more abundantly in Europe, Asia, and North America (Gilbertson and Ryvarden 1986; Wiater et al. 2013). Mostly, this fungus is found growing on decaying logs, stumps, and trunks of many deciduous and coniferous trees (Banik et al. 2010; Wiater et al. 2013). In addition, *L. sulphureus* is known to be a cosmopolitan species, and is also extensively used for manufacturing traditional food with a distinctive flavour (Banik et al. 1998).

A variety of medical activities have been demonstrated by *L. sulphureus* in several therapies. It has been reported that *L. sulphureus* contains various biological active compounds, including alkali-soluble polysaccharides (Olennikov et al. 2009), fatty acids and amino acids content (Agafonova et al. 2007), and laetipolic acid (Davoli et al. 2005). Polysaccharides are the best known, and most potent mushroom-derived substances act as antitumor, anti-HIV, antiviral, antioxidative, antiflammatory, and immunomodulating and hypocholesterolaemic agents (Turkoglu et al. 2007; Zhang et al. 2007; Hwang et al. 2008; Radic et al. 2009). *L. sulphureus* can be used as a good edible mushroom, because the fruiting body contains protein, fibre, carbohydrate, and less fat (Luangharn et al. 2014).

It has been estimated that about 650–700 species of mushrooms belonging to 200 genera are edible (Mortimer et al. 2012); nearly 130 species can be cultivated (Stamets 2000; Boa 2004; Thawthong et al. 2014). In Thailand about 22 mushroom species are commercially grown (Kwon and Thatithatgoon 2004). Wiater et al. (2013) report the first successful pilot scale production of mature fruit bodies of *L. sulphureus* on an artificial sawdust substrate. Several reports have revealed the optimal conditions for mycelia growth of various mushroom species (Shim et al. 2005; Siwulski et al. 2009; Lai et al. 2011). Attempts have been made to investigate the biological activity and mycelia growth of submerged *L. sulphureus* cultures in aqueous media (Davoli et al. 2005; Siljegovic et al. 2011). This study aiming to evaluate the culture conditions for the optimal growth conditions for mycelia growth of Thai strain of *L. sulphureus* is a novel study and no research has been carried out in Thailand so far.

## Materials and methods

### Mushroom collection

Two collections of *L. sulphureus* were collected from a mixed deciduous forest dominated by *Castanopsis* sp. in tropical zone of Chiang Rai, Thailand, and isolated (MFLUCC 12-0546 from dead logs of *Castanopsis* and MFLUCC 12-0547 from living *Castanopsis* tree). Morphological characters of the mushrooms were recorded using the compound microscope (Carl Zeiss™ SteREO Discovery.V8 Microscopes, Jena, Germany).

Pure cultures were isolated from internal tissues into potato dextrose agar (PDA) and incubated at 30°C for 14 days. After incubation, the agar surface was fully covered with a white mycelium. The stock pure culture was covered with mineral oil and deposited at the culture collection of Mae Fah Luang University (MFLUCC) and maintained at 4°C for further study. The nuclear ribosomal internal transcribed spacer region of the mushrooms was sequenced and deposited in GenBank as follows: KM077142 for the strain MFLUCC 12-0546 and KM077143 for the strain MFLUCC 12-0547.

### Effect of media on mycelia growth

Following six different culture media for mycelium growth, namely yeast extract agar (YEA), yeast extract peptone dextrose agar (YEPD), corn meal agar (CMA), oat meal agar (OMA), PDA, and malt extract agar (MEA), were used to determine suitable media for promoting mycelium growth (Table 1). Mycelium morphology on the agar surface was also evaluated. Half a centimetre from the advancing margin of 14 day-old cultures was removed by using a cork borer and placed on the centre of each medium. The Petri dishes were incubated in the dark at 30°C for 10 days. The optimal media for mycelia growth were determined by measuring the colony diameter.

**Table 1.** Composition of various culture media.

	Composition of media (g/L)					
Nutritional reagents	PDA	MEA	YEA	CMA	OMA	YEPD
pH at 25°C	5.6 ± 0.2	5.5 ± 0.2	6.5 ± 0.2	6.0 ± 0.2	7.2 ± 0.2	6.5 ± 0.2
Corn meal infusion from solids				17		
Oatmeal					60	
Meal extract		20				
Dextrose	20	20				20
Peptone		6	5			20
Potato infusion	4					
Yeast extract			3			10
Agar	15	15	15	15	12.5	15

Notes: PDA, potato dextrose agar; MEA, malt extract agar; YEA, yeast extract agar; CMA, corn meal agar; OMA, oat meal agar; YEPD, yeast extract peptone dextrose agar.

### Effect of temperature on mycelia growth

The best medium was used for evaluation at different temperatures (20°C, 25°C, 30°C, and 35°C) to find out the best mycelial growth. The fungal cultures were incubated under dark conditions for 10 days. The colony diameter was measured and compared, and the growth rate was calculated to establish the optimal temperature for mycelia growth of *L. sulphureus*.

### Effect of pH on mycelial growth

The best medium and the optimum temperature were used to evaluate the optimal pH. The pH were adjusted to 5, 6, 7, and 8 with 1 N NaOH or 1 N HCl. The pH range was measured using a digital pH meter before autoclave. The best pH for promoting mycelial growth was determined by measuring the colony diameter.

### Mycelial density

Mycelial growth was determined by using a ruler across the plate and calculated the average of the vertical and horizontal colony diameter. The mycelial density was determined by following Kadiri (1998): + very scanty, 2+ scanty, 3+ moderate, 4+ abundant, 5+ very abundant. The growth rate was calculated by the ratio of colonies diameter and time (days).

### Supplemented cereal grains for spawn production

Five different types of cereal grains and agricultural wastes, namely sorghum, corn cobs, pearl millet, barley grain and sawdust substrate, were used to determine which substrate is the best for spawn production of Thai *L. sulphureus*.

The spawn media were prepared as described by Nwanze et al. (2005). The ingredients included 1 kg of cereal grain/sawdust, 12 g of CaSO_4_ **·**2H_2_O, 3 g of CaCO_3,_ and 1.5 L tap water. The supplemented cereal grain/sawdust was divided into two types (Type 1: all ingredients were mixed with gypsum and CaCO_3_; Type 2: ingredients only). Each cereal grain/sawdust type was washed and soaked overnight, water was drained off, and boiled for 15 minutes, and left to cool down for 20 minutes. Fifty grams of each cereal grain/sawdust were filled into 4-ounce media bottles, autoclaved at 121°C for 15 minutes and left to cool. Before mycelia of *L. sulphureus* were inoculated, the cereal grain/sawdust bottles were shaken to prevent clump formation and to remove 0.5-cm-diameter mycelium discs from the upper side of the spawn media bottles. Spawn bottles were incubated in dark condition at 30°C for 10 days. The linear mycelium length was measured every 2 days for 10 days to calculate the growth rate. All the analyses were carried out in five replicates.

### Statistical analysis

The data of the experiment was analysed by using statistical analysis. The mycelia growth rate values of the last days (10 days) were compared to obtain a mean separation using Tukey’s test (*p = *0.05) followed by post-hoc tests. The results are expressed in a one-way ANOVA analysis using SPSS program (Softonic International SA, Barcelona, Spain).

## Results and discussion

### Mushroom morphology and isolation

**Figure 1.**
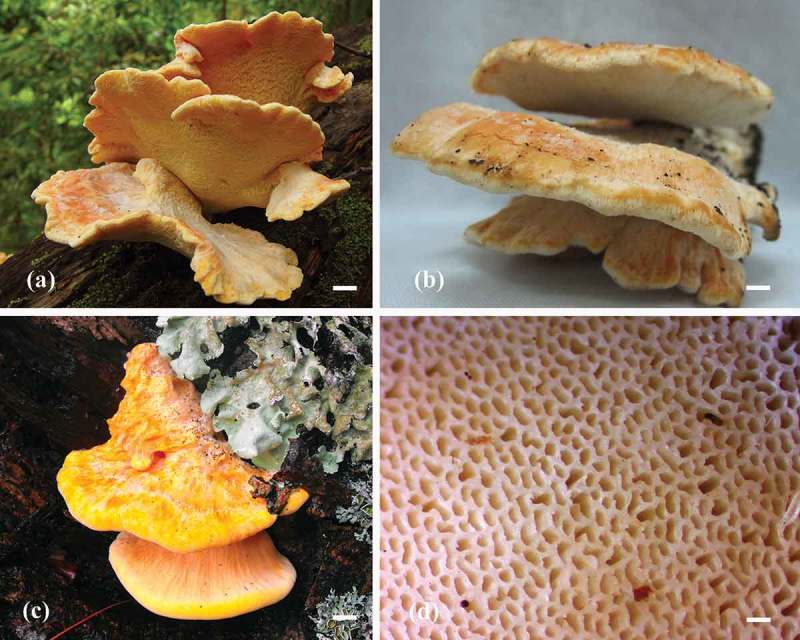
Basidiocarps morphology of *L. sulphureus*: (a), (b) basidiocarps of strain MFLUCC 12-0546; (c) basidiocarps of strain MFLUCC 12-0547; and (d) pore characteristic of *L. sulphureus*. Scale bars: 2 cm (a, b, and c); 500 µm (d).

**Figure 2.**
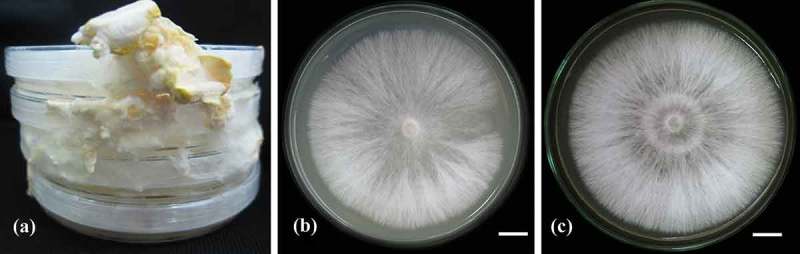
Morphology of *L. sulphureus* mushroom cultures: (a) strain MFLUCC 12-0546 which was incubated at 30°C for 2 months on sterile plastic plate; (b) strain MFLUCC 12-0546 which was incubated at 30°C for 14 days; and (c) strain MFLUCC 12-0547 which was incubated at 30°C for 14 days. Scale bars: 1 cm (b and c).

Basidiocarps of *L. sulphureus* vary considerably in size (7–14 cm wide and 12–30 cm across). The distinctive characteristics of the fruiting bodies are the light yellow-dark orange colour, bracket clusters, thick-soft surface when freshly and chalky character when dry (see Figure 1(a)–(c)). The pores of the basidiocarps are pale yellow in colour (see Figure 1(d)). The internal tissues of *L. sulphureus* fruiting bodies were cut and placed on PDA plates and incubated at 30°C. After 2 months, the mycelia were fully covered the plates and produced young primodial fruiting bodies (see Figure 2(a)), observed after incubating the fully mycelia covered PDA plates for 10 days (see Figure 2(b) and 2(c)). These stock pure cultures are maintained in PDA slant tubes at 4°C while with 15% glycerol on the top of the PDA slants at −20°C for long-term preservation, and were deposited at Mae Fah Luang University culture collection (voucher specimen no. MFLUCC 12-0546 and MFLUCC 12-0547).

**Figure 3.**
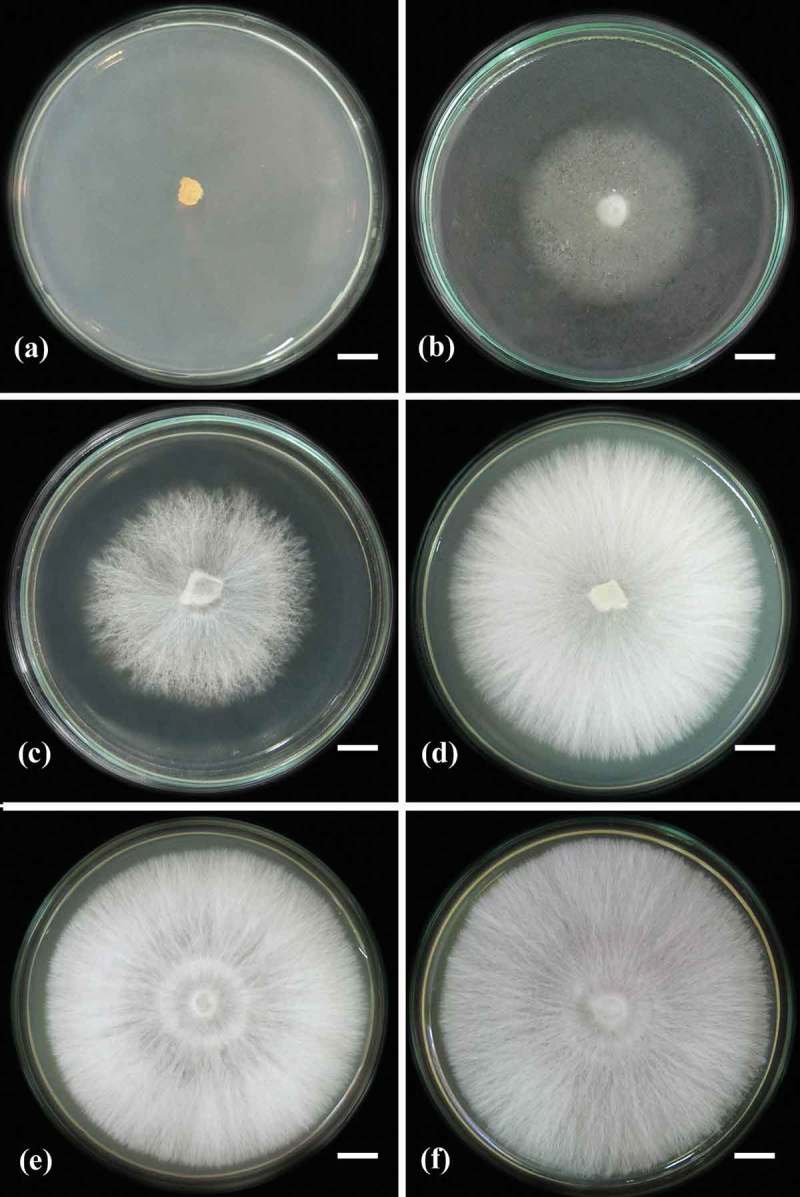
Mycelia growth of *L. sulphureus* strain MFLUCC 12-0546 on various solid media which were incubated at 30°C for 10 days: (a) YEPD; (b) YEA; (c) CMA; (d) OMA; (e) PDA; and (f) MEA. Scale bars: 1 cm (a to f).

**Figure 4.**
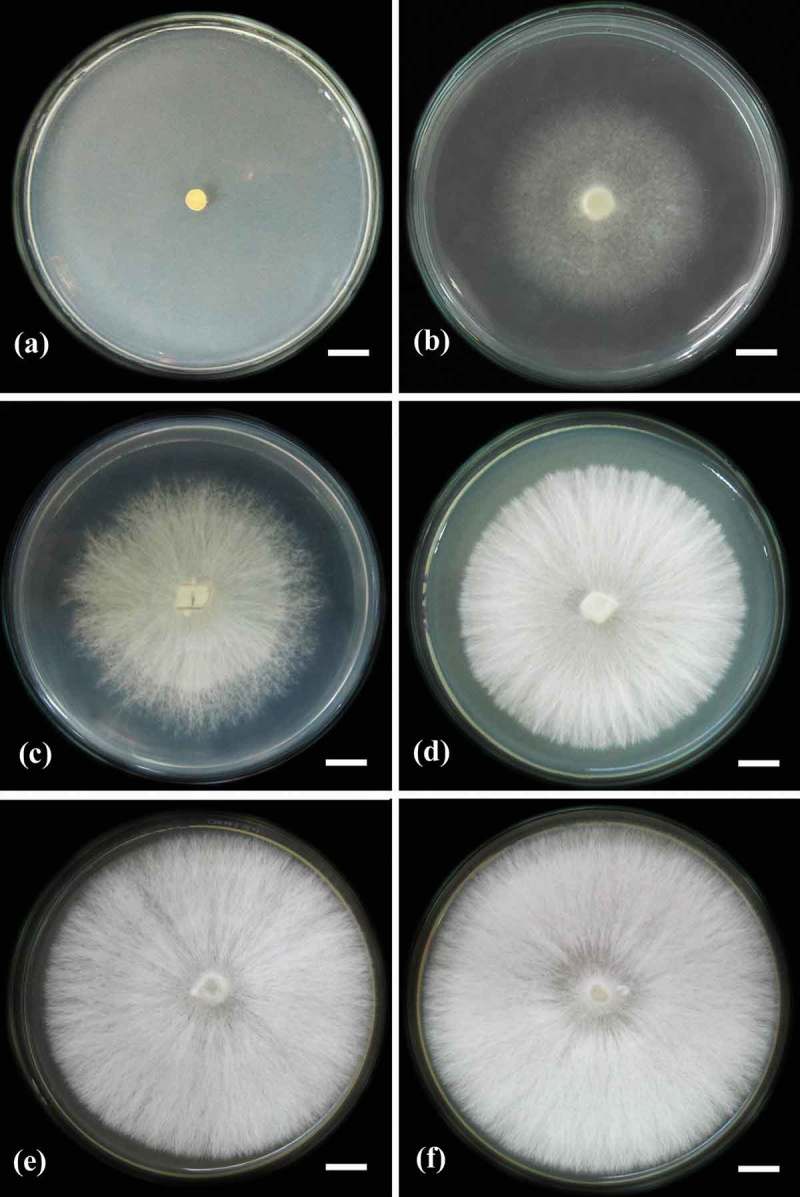
Mycelia growth of *L. sulphureus* strain MFLUCC 12-0547 on various solid media which were incubated at 30°C for 10 days: (a) YEPD; (b) YEA; (c) CMA; (d) OMA; (e) PDA; and (f) MEA. Scale bars: 1 cm (a to f).

### Effect of media on mycelial growth

Siwulski et al. (2009) has discussed one of the first reports of the influence of solid media for *L. sulphureus* mycelia growth. In our study, six different solid media were screened for the favourable growth of both Thai *L. sulphureus* strains. The best mycelium colony diameter was observed on PDA and MEA media, followed by CMA, OMA, and YEA, respectively.

*L. sulphureus* strain MFLUCC 12-0546 had the largest colony diameter on PDA medium (1.08 cm/days), density (4+); MEA medium (1.06 cm/day), density (4+); and CMA (0.95 cm/day), density (3+) (Figure 3). Strain MFLUCC 12-0547 had the largest mycelial colony diameter on PDA medium (1.10 cm/days), density (4+) and MEA medium (1.00 cm/day), density (4+) (Figure 4), while it did not grow on YEPD medium at all (Table 2). Our results are similar to those of Siwulski et al. (2009). *L. sulphureus* grew well on PDA medium.

**Table 2.** Effect of various solid media on mycelial growth (cm) and mycelial growth rate (cm/day) of *L. sulphureus* strains MFLUCC 12-0546 and MFLUCC 12-0547. Values with the same letter are not significantly different (*p < *0.05) by the Tukey’s test.

	Colony diameter		Growth rate		Mycelial density	
	MFLUCC	MFLUCC	MFLUCC	MFLUCC	MFLUCC	MFLUCC
Culture media	12-0546	12-0547	12-0546	12-0547	12-0546	12-0547
PDA	1.08 ± 0.04^a^	1.10 ± 0.05^a^	1.08	1.10	4+	4+
MEA	1.06 ± 0.05^a^	1.00 ± 0.12^a^	1.03	1.00	4+	4+
YEA	0.60 ± 0.06^c^	0.55 ± 0.02^b^	0.60	0.55	2+	2+
CMA	0.95 ± 0.12^ab^	0.93 ± 0.07^a^	0.94	0.93	3+	3+
OMA	0.75 ± 0.24^bc^	0.67 ± 0.17^b^	0.74	0.67	5+	5+
YEPD	0.00^c^	0.00^c^	0.00	0.00	–	–

Notes: PDA, potato dextrose agar; MEA, malt extract agar; YEA, yeast extract agar; CMA, corn meal agar; OMA, oat meal agar; YEPD, yeast extract peptone dextrose agar.

### Effect of temperature on mycelial growth

Two Thai *L. sulphureus* strains were tested for the suitable temperature for promoting mycelial growth on MEA medium. Temperatures of 20°C, 25°C, 30°C and 35°C were used and the mycelial grew well between 25°C and 30°C, while the most unfavourable was 35°C (Table 3). Even though the mycelial growth of *Macrolepiota procera* (Scop.:Fr) Singer and *Lignosus rhinoceros* (Cooke) occurred at 30°C (Shim et al. 2005; Lai et al. 2011), growth of *Ganoderma lucidum* was observed at 25**–**30°C (Jayasinghe et al. 2008). The optimal temperature for Thai *L. sulphureus* was related to a comment did by Mswaka and Magan (1999), ‘the optimal temperature for wood-decay fungi from temperate regions is between 25°C and 30°C’; thus, we consider the suitable temperature for Thai *L. sulphureus* between 25°C and 30°C.

**Table 3.** Mycelial growth rate (cm/day) of *L. sulphureus* strain MFLUCC 12-0546 and strain MFLUCC 12-0547 at different temperatures. Values with the same letter are not significantly different (*p < *0.05) by the Tukey’s test.

Temperature (°C)	Mycelial growth rate	
	MFLUCC 12-0546	MFLUCC 12-0547
20	0.64 ± 0.06^c^	0.82 ± 0.07^b^
25	1.22 ± 0.04^a^	1.15 ± 0.04^a^
30	0.81 ± 0.04^b^	1.03 ± 0.06^ab^
35	0.00^d^	0.00^d^

### Effect of pH for mycelial growth

All pH from 5–8 were suitable for growth of mycelium of *L. sulphureus*. The optimal pH on mycelia growth of *L. sulphureus* was in the range of pH 6–8, however (Table 4). The optimal pH for *Macrolepiota procera, Lignosus rhinoceros*, and Thai Oyster mushroom were 5–8 (Shim et al. 2005; Lai et al. 2011; Kumla et al. 2013).

**Table 4.** Mycelial growth rate (cm/day) of *L. sulphureus* strains MFLUCC 12-0546 and MFLUCC 12-0547 at different pH. Values with the same letter are not significantly different (*p < *0.05) by the Tukey’s test.

pH	Mycelial growth rate	
	MFLUCC 12-0546	MFLUCC 12-0547
5	0.84 ± 0.06^b^	0.84 ± 0.06^b^
6	0.98 ± 0.10^ab^	0.99 ± 0.07^ab^
7	1.05 ± 0.06^a^	1.02 ± 0.06^a^
8	1.04 ± 0.03^a^	1.05 ± 0.03^a^

### Supplemented cereal grains/sawdust for spawn production

Our study examined the use of different cereal grain/sawdust media for promoting mycelia growth and spawn production. After 10 days of incubation, *L. sulphureus* mycelium was able to colonize all the cereal grains and agricultural waste medium (sawdust). The data for mycelium growth on different cereal grain media/sawdust was investigated and is shown in Table 5. If we consider type 1, the highest mycelial growth was obtained on pearl millet (0.45 cm/day) and sorghum grains (0.44 cm/day), for strain MFLUCC 12-0546 and strain MFLUCC 12-0547 (0.55 cm/day) on pearl millet, followed by corn cobs, barley grains, and sawdust, respectively. For type 2, the strain MFLUCC 12-0546 grew well on sorghum grains (1.01 cm/day), and MFLUCC 12-0547 grew well on pearl millet (1.16 cm/day) and sorghum grains (1.00 cm/day). Therefore, the results showed that the various cereal grain media can be used in order to promote mycelia growth of *L. sulphureus*. In general, most of the commercial traditional spawns are also made using sorghum grains as the substrate, because of low cost and availability (Ogden and Prowse 2004). Sorghum grain was the best substrate for spawn production for Thai Oyster mushroom cultivation (Kumla et al. 2013). In addition, it is advised to use the undamaged cereal grains, as the broken grains are more prone to spawn contamination (Narh et al. 2011).

**Table 5.** Effect of different types of cereal grain media and sawdust on mycelia growth rate (cm/day) of *L. sulphureus*. Values with the same letter are not significantly different (*p < *0.05) by the Tukey’s test.

	Type 1		Type 2	
	MFLUCC	MFLUCC	MFLUCC	MFLUCC
Cereal grain media	12-0546	12-0547	12-0546	12-0547
*Sorghum bicolor* L. (Sorghum)	0.44 ± 0.08^a^	0.44 ± 0.08^ab^	1.01 ± 0.06^a^	1.00 ± 0.06^a^
*Zea mays* (Corn cobs)	0.34 ± 0.05^ab^	0.35 ± 0.02^ab^	0.26 ± 0.08^c^	0.25 ± 0.06^c^
*Pennisetum glaucum* (Pearl millet)	0.45 ± 0.04^a^	0.55 ± 0.02^a^	0.69 ± 0.20^b^	1.16 ± 0.03^a^
*Hordeum vulgare* L. (Barley)	0.35 ± 0.10^ab^	0.33 ± 0.08^ab^	0.43 ± 0.10^c^	0.42 ± 0.07^c^
Sawdust	0.27 ± 0.01^b^	0.25 ± 0.01^b^	0.43 ± 0.11^c^	0.42 ± 0.10^c^

Notes: Type 1, all ingredients were mixed with gypsum and CaCO_3_; Type 2: ingredients only.

## Conclusion

In this study, we carried out experiments to investigate the optimal conditions for promoting mycelial growth of *L. sulphureus* from tropical northern Thailand. The favourable media for mycelia growth were PDA and MEA, while the favourable temperature and pH were 25–30ºC and 6–8, respectively. In addition, both strains grew well on sorghum grains, and the highest mycelia growth was showed on sorghum, pearl millet, and barley grains when mixed throughly with gypsum and CaCO_3_. Since this is the first study of optimal conditions on mycelia growth of Thai *L. sulphureus*, we would like to recommend PDA and MEA as culture media under temperature 25–30ºC and pH 6–8, and sorghum as the spawn medium, for growing *L. sulphureus* in Thailand. There is a very high chance to domesticate local Thai strains of *L. sulphureus*, considering the results of our study and the study of Wiater et al. (2013).

## Funding

This work was financially supported by the Bioresource Research Network (BRN) under the project ‘Value added products from basidiomycetes: Putting Thailand’s biodiversity to use’ [grant number BRN049/2553]; the National Research Council of Thailand (NRCT) project ‘Taxonomy, phylogeny and cultivation of *Lentinus* species in northern Thailand’ [grant number NRCT/55201020007]; Mae Fah Luang University project ‘Taxonomy, phylogeny and cultivation of *Lentinus* species in northern Thailand’ [grant number MFU-54101020048]; and Thailand Research Fund grant ‘Taxonomy, phylogeny and biochemistry of Thai Basidiomycetes’ [grant number BRG 5580009].
